# Curcumin in Wound Healing—A Bibliometric Analysis

**DOI:** 10.3390/life13010143

**Published:** 2023-01-04

**Authors:** Faiza Farhat, Shahab Saquib Sohail, Farheen Siddiqui, Reyazur Rashid Irshad, Dag Øivind Madsen

**Affiliations:** 1 Department of Zoology, Aligarh Muslim University, Aligarh 202002, India; 2 Department of Computer Science and Engineering, Jamia Hamdard University, New Delhi 110062, India; 3 College of Science and Arts, Najran University, Najran 11001, Saudi Arabia; 4 School of Business, University of South-Eastern Norway, 3511 Hønefoss, Norway

**Keywords:** curcumin, curcuma longa, turmeric, wound healing, bibliometric analysis

## Abstract

Background: Curcumin has been widely used to treat a variety of diseases and disorders since ancient times, most notably for the purpose of healing wounds. Despite the large number of available reviews on this topic, a bibliometric tool-based meta-analysis is missing in the literature. Scope and approach: To evaluate the influence and significance of the countries, journals, organizations and authors that have contributed the most to this topic, the popular bibliometric markers, including article count, citation count, and Hirsch index (H-index), are taken into account. Their collaborative networks and keyword co-occurrence along with the trend analysis are also sketched out using the VOSviewer software. To the best of our knowledge, this is the first bibliometric review on the topic and hence it is envisaged that it will attract researchers to explore future research dimensions in the related field. Key findings and conclusions: India provided the most articles, making up more than 27.49 percent of the entire corpus. The International Journal of Biological Macromolecules published the most articles (44), and it also received the most citations (2012). The Journal of Ethnopharmacology (28 articles) and Current Pharmaceutical Design (20 articles) were the next most prolific journals with 1231 and 812 citations, respectively. The results indicate a significant increase in both research and publications on the wound-healing properties of curcumin. Recent studies have concentrated on creating novel medicine-delivery systems that use nano-curcumin to boost the effect of the curcumin molecule in therapeutic targeting. It has also been observed that genetic engineering and biotechnology have recently been employed to address the commercial implications of curcumin.

## 1. Introduction

The use of natural products against sickness and affliction has only recently caught the attention of the scientific community, despite the fact that many plant-derived compounds have been utilized for the treatment of various ailments since antiquity. In comparison to manufactured pharmaceuticals, medicinal plants are thought to be better in terms of side effects, easy accessibility, and availability at little or no cost. This has led to widespread use of numerous plants and their derivatives in the Asian and African subcontinents. The effectiveness of therapeutic herbs such as *Allium sativum* (garlic) [1], *Piper longum* [2], *Azadirachta indica* (neem) [3], *Nigella sativa* (black cumin) [4], *Curcuma longa* (turmeric) [5], etc., have been tested against a number of diseases. Among all the plant derivatives, curcumin, an active ingredient of *Curcuma longa*, has been frequently used against a multiple array of diseases including wound healing [6]. The spice *Curcuma longa* is widely used throughout the world, including India. It has a long history of use in medicine, with the earliest known evidence dating back 6000 years [7]. Curcumin is a naturally occurring polyphenol that can be found in the rhizome of *Curcuma* species [8], and it has a variety of properties including antimicrobial, antioxidant, and anti-inflammatory activities, making it particularly useful in the process of healing wounds [9]. 

### 1.1. Curcumin as a Wound-Healing Agent

Wound healing is a very complex natural process involving several interconnecting steps to restore the normal structural integrity and function of the damaged area. The whole process of wound healing is divided into four phases: homeostasis, inflammation, proliferation, and tissue remodeling [10]. An ideal wound-healing agent should have antimicrobial properties to keep the wound from getting infected, anti-inflammatory properties to lessen inflammation, and proliferative and regenerative properties to speed up the process of cell proliferation and tissue remodeling [11]. Curcumin has been used to treat wounds since ancient times, but only recently have a number of studies provided scientific evidence of its effectiveness in treating both acute and chronic wounds [12]. In addition to being antibacterial [13], antioxidant, and anti-inflammatory, curcumin also promotes tissue proliferation and remodeling, making it a highly potent healing agent. The importance of curcumin in every stage of wound healing has been established by recent investigations. By preventing the generation of two key cytokines that are crucial to inflammation, interleukin-1 (IL-1) and tumor necrosis factor alpha (TNF-), it controls the inflammatory responses [14]. Additionally, it dramatically lowers the expression of antioxidant enzymes, one of the primary causes of inflammation and the oxidation process [15]. It also increases fibroblast migration, granulation tissue development, collagen deposition, and overall re-epithelization to improve proliferation. Furthermore, by boosting TGF- synthesis and consequently fibroblast proliferation, it encourages tissue remodeling and wound contraction [1].

### 1.2. Reviews on Curcumin and Its Wound-Healing Properties

A number of bibliometric reviews on curcumin in general have been published recently [16,17,18], reflecting the importance of curcumin in current research trends. Several reviews on curcumin and its therapeutic benefits have been published, incorporating what is already known and demonstrating the development of this particular field of research. These reviews, however, suffer from the following limitations. First, the majority of them are either brief reviews or systematic reviews [19,20,21,22,23]. Second, despite being an increasingly significant and dynamic multidisciplinary research area, very few publications specifically address the wound-healing abilities of curcumin. Furthermore, for quantitative reviews on this specific subject, no bibliometric methodologies have been adopted to date. 

This study offers a quantitative perspective based on bibliometric data in light of the significance of the research on curcumin as a potential wound-healing agent. Bibliometric analysis has long been acknowledged as a crucial instrument in the evaluation of scientific output by enabling researchers to compile and evaluate works in their areas of interest [24]. Bibliometric analysis is utilized for a variety of purposes, including identifying new patterns in the performance of articles and journals, research keywords, collaboration networks, and the assessment of the particular topic in the body of existing literature. By examining the interpersonal connections between various authors, countries and institutions, bibliometric analysis describes the bibliometric and intellectual framework of a particular field of interest [25]. Based on our search and to the best of our knowledge, this is the first bibliometric review on the topic; hence, it is envisaged that it will encourage researchers to explore future research dimensions in the related field. The aim of the present study is to present a bibliometric analysis of the recent literature concerning the wound-healing properties of curcumin. To achieve the aim of the study, the data is analyzed using standard parameters such as the annual publication, contributing journals, countries, institutions and authors using SCOPUS analysis, VOSviewer and MS Excel software. Additionally, bibliometric analysis of keyword co-occurrence is also addressed using VOSviewer software. 

## 2. Results and Discussions:

### 2.1. Trend Analysis of Publications

**Figure 1:**
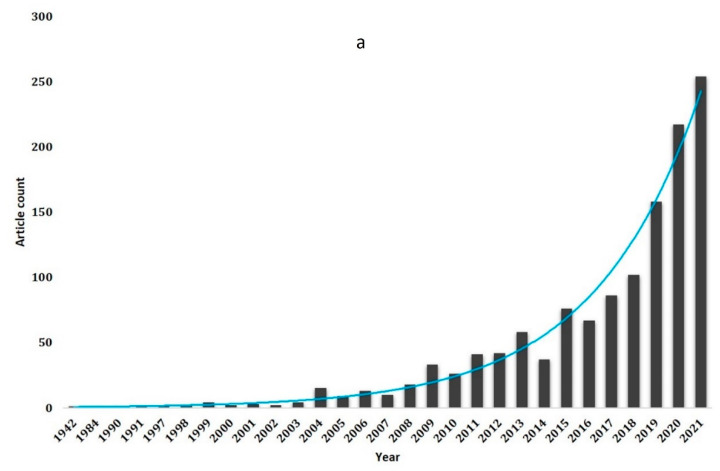

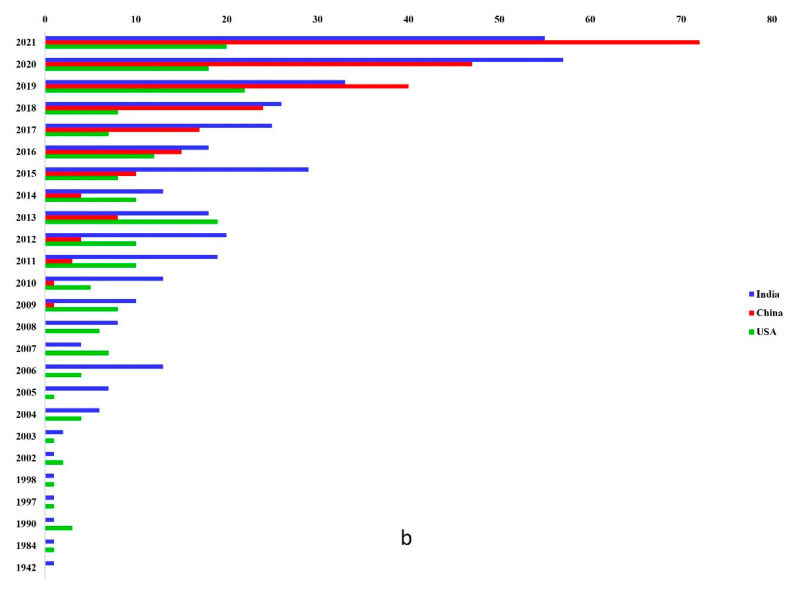
(**a**). Trend analysis of publications per year showing an exponential growth in annual publications through time. (**b**). Comparative trend analysis of the top 3 contributing countries viz., India, China and USA.

The article trends published between 1942 and 2021 are shown in Figure 1a. Until 2003, there was an average of only 2 articles published annually on the topic of curcumin in wound healing. The first one was published in 1942. From 2004 to 2012, the annual publications exhibited a relatively sluggish growth, with an average release of up to 23 articles/year. In 2013, the number of publications surpassed 50, which can be considered an inflection point. Afterwards, the distribution of publications was not continuous, but the data revealed a significant increase, especially from 2013 onward, with more than 82.16 percent of the papers produced after 2012. Such a development profile illustrates how this field is becoming more and more influential. After 2018, the annual publishing doubled with an average of 117 articles per year, reaching its peak in 2021 (254 articles/year). Based on the pattern of development observed, we can infer that curcumin research in wound healing and its application will continue to garner more and more attention in academia in the near future.

The rapid growth in publications from 1942 to 2021 is obvious from the diagram, and a trend analysis of the annually generated corpus from the top 3 contributing countries—India, China, and the United States—is more interesting. Although the trend is consistent, the annual production is highly unpredictable. Up until 2005, India and the USA had a slow rise, but after that, a significant increase in publication was noted. India outpaces the US in terms of article production, reaching up to 55 articles annually compared to the US’s maximum of 22. China, on the other hand, began publishing on the chosen study topic only in 2006 and has already surpassed India in terms of publications/year (72 articles/year), claiming second place in corpus output (Figure 1b). Moreover, exponential regression analysis suggests that India and USA growth can be fitted to$\;y={\left(1.9126\;*\;1.0470\right)}^{x}$ and$\;y={\left(1.6413\;*\;1.0357\right)}^{x}$ respectively. As far as China is concerned, research in the topic under consideration was initiated in 2009, after which China also witnessed an exponential growth with$\;y={\left(0.6286\;*\;1.1311\right)}^{x}$.

### 2.2. Analyses of Journals

The 1284 publications are dispersed among 159 various journals, demonstrating the variety of sources on the subject of curcumin and its potential to heal wounds. The top 10 journals in terms of the article count on the use of curcumin for wound healing were responsible for 14.48 percent of the corpus (Table 1) and 16.07 percent of the total citation count. The *International Journal of Biological Macromolecules* stands out as the most prolific publication source, having the most articles published (44) and also the most citations (2012), followed by the *Journal of Ethnopharmacology* (28 articles) and *Current Pharmaceutical Design* (20 articles), with 1231 and 812 citations, respectively. The *International Journal of Biological Macromolecules* also has the most cited article, with 489 citations. With 307 citations, the second-most cited article is one from *Phytotherapy Research*. If we look at the top journals by H-index (according to https://www.scimagojr.com/journalrank.php), the *International Journal of Pharmaceutics* appeared in first place with an H-index of 229. The *Journal of Ethnopharmacology* came in second with an H-index of 205 (195). When comparing annual output, the majority of journals including the *International Journal of Biological Macromolecules*, *Current Pharmaceutical Design*, and the *International Journal of Molecular Sciences* demonstrated a growth in this particular field of research. 

### 2.3. Analyses of Contributing Countries

The 1284 articles were contributed by 98 different nations/regions overall. Table 2 shows the top 10 countries measured by article count, among which India ranked first, followed by China, USA, Iran, and Italy. From an article count perspective, India contributed the most articles (354 articles), participating in more than 27.49 percent of the total corpus which is likely due to the high usage of curcumin products in Indian subcontinent. China (18.76%—240 articles) ranked the second in highest contribution, followed by the USA (12.77%—164 articles), Iran (8.64%—111 articles), and Italy (3.5%—45 articles). Additionally, the USA exhibits a stronger academic impact than the rest of the globe, as seen by the highest number of citations (13,361). In terms of citations, India is second (11,361). Furthermore, with citation counts of 6346, 3068, 1867, and 1615, respectively, technologically advanced nations including China, Iran, Italy, and South Korea have made major contributions to this topic. When annual productivity was compared, most countries/regions showed a positive surge in interest in the research field, for example, in India, USA, and China. 

### 2.4. Analyses of Contributing Authors and Institutions

A total of 5503 authors contributed to the 1284 articles. However, just three and seven authors, respectively, contributed to more than seven and not fewer than five papers. Table 3 lists the top 10 authors according to the number of articles they have published on the chosen study topic. Meiyanto, Edy is placed first with 9 articles, followed by Sahebkar, Amirhossein and Jenie, Riris with 8 and 7 articles published, respectively, on curcumin as a wound-healing agent. The top 5 authors ranked by their H-index were Aggarwal B.B.; Ramakrishna, Seeram; Sahebkar, Amirhossein; Maheshwari, R.K. and Hussain, Zahid. 

The 1284 publications included participation from 3658 institutions in total. However, only 4 and 22 institutions, respectively, participated in more than 20 and 10 articles. Among the top 5 institutions as determined by article count, Mashhad University of Medical Sciences participated in the most articles, followed by Shahid Beheshti University of Medical Sciences, the Ministry of Education of China, and the Central Leather Research Institute of India (Table 4). The majority of institutions, including Shahid Beheshti University of Medical Sciences, the Ministry of Education of China, and the Central Leather Research Institute of India, demonstrated an increase in interest in the field when comparing the annual productivity.

### 2.5. Collaboration Analysis

The substantial collaboration among academics and researchers at all levels is a significant and distinctive aspect of academic research. An effective metric for assessing research collaboration is the level of academic cooperation [26]. The current study was conducted to evaluate authors, institutions, and nations in order to gauge the level of academic collaborations among them.

**Figure 2:**
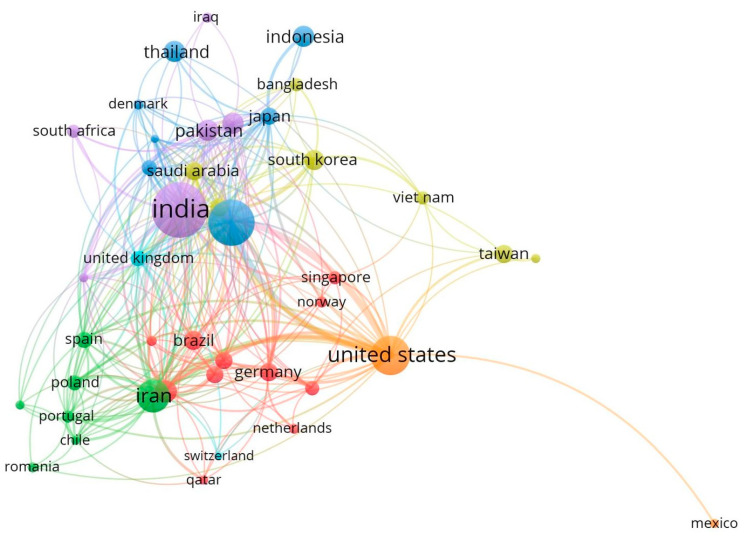
Collaboration network of contributing countries. The size of the circle is directly related to the documents that each country generated, and the fine lines demonstrate the collaboration strength. When it comes to collaboration networks, the United States came out on top with the most (33 countries) in the world. With 32 collaborators, India came in second position, ahead of Iran (29), Italy (27), and China (23).

The collaboration analysis among contributing countries revealed 43 productive countries with at least five publications each (Figure 2). The size of the circle is directly related to the documents that each country generated, and the fine lines demonstrate the collaboration strength. In terms of article counts, India came in front with the largest circle, followed by China, the United States, and so forth. With regards to collaboration networks, the United States came out on top with the most (33 countries) in the world. With 32 collaborators, India came in second position, ahead of Iran (29), Italy (27), and China (23). In 23 publications, China placed first among the US’s collaborating partners, followed by India (22) and Iran (18). India and Saudi Arabia (10) and India and South Korea (7) were the second-most frequent collaborators.

**Figure 3:**
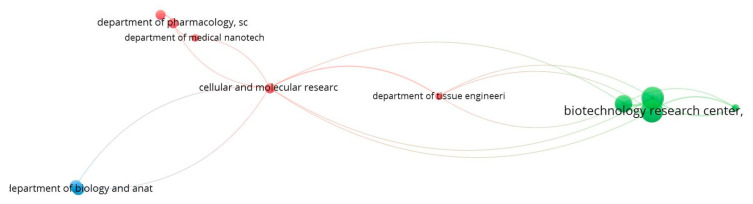
Collaboration network of institutions. The Cellular and Molecular Research Center of Iran University of Medical Sciences is the most collaborative, with 8 different collaborators followed by Biotechnology Research Center (5), Neurogenic Inflammation Research Center (5) and School of Pharmacy (5).

When the collaboration analysis of research institutions is visualized in VOSviewer, 11 prolific institutions appeared, organized in 3 clusters (Figure 3). The red cluster includes five research institutions out of which three institutes were from *Tehran University of Medical Sciences* (*Experimental Medicine Centre*, *Department of Pharmacology* and *Department of Medical Nanotechnology*) and two institutes were from *Iran University of Medical sciences* (*Cellular and Molecular Research Center* and *Department of Tissue Engineering and regenerative medicine*). The green cluster consists of four research institutions, out of which three are from *Mashhad University of Medical Sciences* (*Biotechnology Research Center*, *Neurogenic Inflammation Research Center* and *School of Pharmacy*) and *Halal Research Centre*, *Iran*. The *University of Qom* and *Shahid Beheshti University of Medical Sciences* comprise the blue cluster. The *Cellular and Molecular Research Center* of *Iran University of Medical Sciences* is the most collaborative, with 8 different collaborators followed by *Biotechnology Research Center* (5), *Neurogenic Inflammation Research Center* (5) and the *School of Pharmacy* (5). 

**Figure 4:**
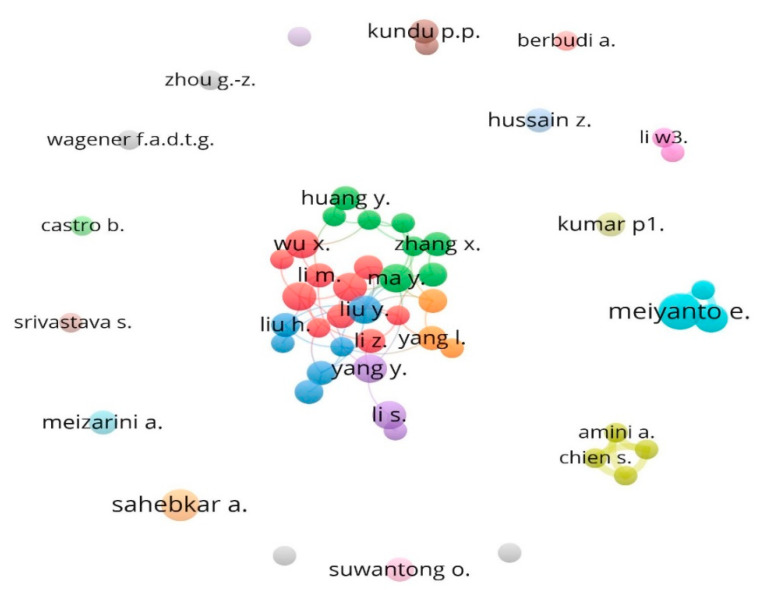
Collaboration network of authors. Amini, A., Chien, S., Ghoreishi, S.K. and Bayat, M. are the closest partners with the highest link strength of 15 among each other. Meiyanto, E., and Jenie, R.I. are the second most collaborative partners with a link strength of 12.

For the analysis of the collaborating network of authors, it was found that there were 30 prolific authors organized in 22 clusters, with an overall link strength of 115 (Figure 4). These categories show how closely the different authors collaborate. The total link strength indicates the total strength of the co-authorship links of a given researcher with other researchers. Amini, A., Chien, S., Ghoreishi, S.K. and Bayat, M. are the closest partners with highest link strength of 15 among each other. Meiyanto, E., and Jenie, R.I. are the second most collaborative partners with a link strength of 12, followed by Putri, H. and Liu, Y with link strength of 10. Liu Y. was the most collaborative author, collaborating with 9 other authors, followed by Ma Y. and Wang S. with 7 collaborating authors. 

### 2.6. Keyword Co-Occurrence Analysis

**Figure 5:**
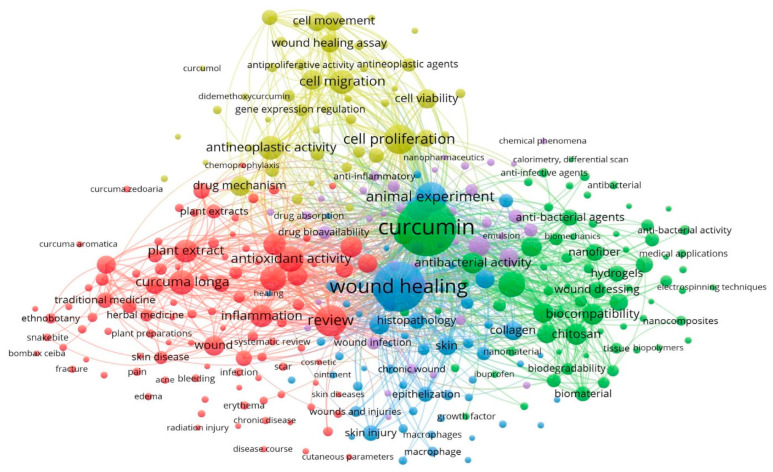
Keyword co-occurrence analysis.

The study of co-occurrence of keywords provides a summary of the research terms used in the corpus and indicates the direction of recent research trends [25]. When the gathered data is imported into the VOSviewer software, a total of 16,208 keywords appeared. Keywords relevant to the wound-healing applications of curcumin and having occurrences greater than 10 were retained, and the rest were removed. As a result, 320 keywords remained, arranged into 5 clusters with 23,481 links (Figure 5). The red cluster consists of the keywords “curcuma longa” and terms such as “herbal medicine” “traditional medicine” “plant extract”, as well as terms for skin conditions such as “skin illness”, “acne bleeding”, “erythema” and “edema”. The terms “cell proliferation”, “antineoplastic activity”, “anti-inflammatory”, and “gene expression regulation” are among those found in the yellow cluster and are all related to the properties of curcumin. Keywords such as “nanocomposite”, “nanofibre”, “nano material”, “biomechanics”, “hydrogel”, “emulsion” and “electrospinning techniques” are found in the green cluster, which is devoted to biotechnology and drug delivery mechanics. The blue cluster of keywords is connected with skin infection and includes the terms “wound infection”, “wound healing”, “histopathology”, “chronic wound”, “wound and injuries”, “epithelization” and “skin injury”.

**Figure 6:**
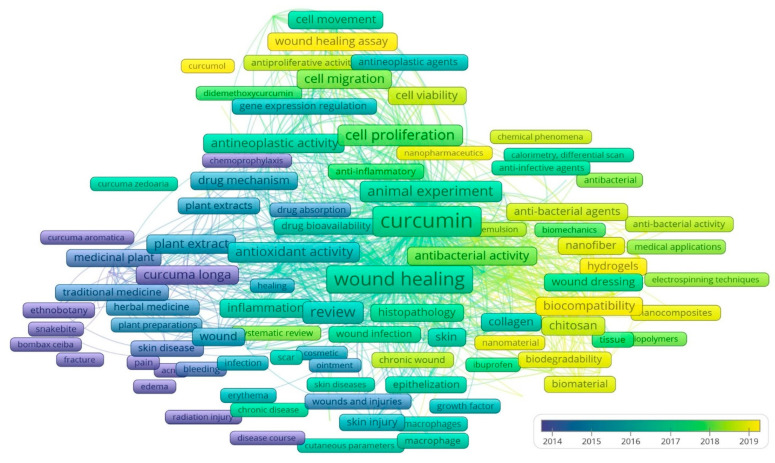
Evolution of keyword co-occurrence through time. Topics shown in blue were prevalent at the beginning of the year 2000, green topics were more prevalent between 2015 and 2018, and yellow topics predominated in the years following 2018.

The overlay visualization analysis shows the evolution of keywords over time (Figure 6). Topics shown in blue were prevalent at the beginning of the year 2000, green topics were more prevalent between 2015 and 2018, and yellow topics predominated in the years following 2018. While early topics focused on conventional medical uses of curcumin in wound healing, more current issues pertain to genetic engineering and biotechnology to improve new medication delivery systems. It has been noted that “curcumin” as a word has only been used since 2015, while “curcuma longa” and “curcuma” have been used for a very long time. Therefore, we have chosen our search string accordingly, including “turmeric” as well, in order to obtain the previous studies. We also used the search string “cur-cum*,” which ensures that curcumin as well as curcuma longa are included in the results. The frequent co-occurrence of keywords such as “wound,” “skin injury”, “edema”, “skin diseases”, “erythema”, “dermatitis”, “burn”, “scar” and “wounds and injuries” from the very beginning proves that curcumin has been used in treating various skin ailments since ancient times. However, since 2015, “wound healing” has been used more frequently (702 times), making it the second most commonly occurring terminology after curcumin (922 occurrence). The recent research has been more focused on drug delivery systems using modern biotechnological techniques to enhance the specificity of the curcumin molecule toward the drug target. This is evident from the sharp rise of keywords such as “electrospinning techniques”, “hydrogels”, “biocompatibility”, “biomechanics”, “chitosan”, “nano pharmaceutics”, “nanocomposites”, “emulsion” and “biomaterial”.

The top 10 trending keywords identified from the overlay visualization of keywords include “curcumol”, “hydrogels”, “nanocomposites”, “wound healing assay”, “nanomaterial”, “nano pharmaceutics”, “biocompatibility”, “nanofiber”, “chitosan” and “biomaterial”. These keywords not only demonstrate the current research directions but also suggest that a variety of techniques are being employed to use curcumin in healing acute as well as chronic wounds. 

**Figure 7:**
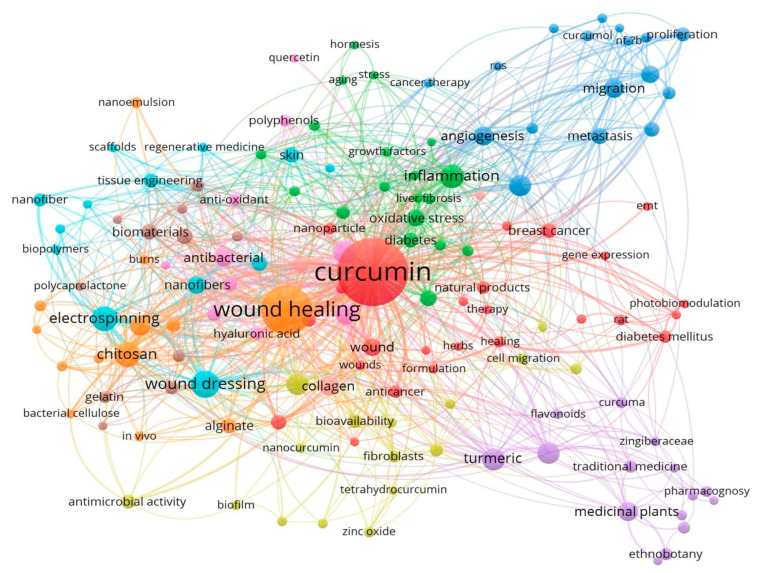
Author’s keyword co-occurrence.

When the co-occurrence analysis of the author’s keyword was conducted, a total of 3143 keywords appeared (Figure 7). It is noted that the keywords chosen by the contributing authors are quite diverse, ranging from traditional keywords such as “curcuma longa”, “turmeric” and “medicinal plant” to keywords for modern integrated technologies to evaluate the effectiveness of curcumin bioavailability, such as “nanoparticles”, “drug delivery”, “nano emulsion”, tissue engineering and “nanofibres”. Additionally, “curcumin” and “wound healing” rank as the top two keywords in both the author’s keyword and the overall keyword list. The top 20 total keywords as well as the author’s keyword with co-occurrences are listed in Table 5. 

Herbal treatments have been used to heal wound infections since ancient times. The active component of the therapeutic herbs, however, is now the subject of investigation. One of the most investigated substances with pleiotropic effects in treating skin disorders is curcumin, the active ingredient of the medicinal plant *Curcuma longa*. Bibliometric assessments can assist to forecast future trends in a certain academic topic by providing a high-level overview of its current state [27]. Therefore, in this study, we investigated publications on curcumin and wound healing with a focus on the countries, organizations, journals, authors, and trending keywords related to this subject. A total of 1284 journal articles, available in the SCOPUS database, were published from 1942 to 2019. The bibliometric data revealed that as of 2019, 5503 active authors from 98 different countries had contributed to this area of research. There were 3658 organizations involved in publishing these articles in 159 different journals. It was found that curcumin is one of the trending molecules in terms of wound-healing applications. A tremendous rise in this area of research represents the increasing interest in wound-healing applications of curcumin. Wound-healing research has made significant strides, and it is an active and quickly growing field of scientific interest. Here, we discovered that over 98 countries have made contributions to the field and the number of papers devoted to this topic is increasing every year. In light of these findings, we predict that numerous in-depth research examining curcumin and its uses in wound healing will be published in the upcoming years. 

The academic influence of a country can be assessed by the total number of citations it receives. The United States has a greater academic influence than the rest of the world, as evidenced by the highest number of citations (13,361), despite the fact that India provided the most articles (354 articles), accounting for more than 27.49 percent of the total corpus pertaining to curcumin and wound healing. Although the USA now leads in the number of citations in this field, India and China have significantly more publications than the USA, and the quantity and quality of studies undertaken in these two countries are constantly increasing, positioning it to overtake the USA soon. This is apparent from the fact that, while having much lower average citation rates per article than the United States (81.46), India (32.09) and China (26.44) are ranked second and third overall in terms of total citation counts. One possible reason for this trend could be the strict rules governing the use of natural compounds like curcumin in USA. The development of globally accepted natural products is difficult due to diverse regulatory standards for product safety, quality, and efficacy, as well as disparities in the status of components and excipients, which are impeding the growth of the herbal drug sector. The claims have to abide by the FDA’s regulatory requirements [28,29,30]. In contrast to this, India has encouraged the use of natural products like curcumin for different therapies, especially Ayurvedic treatment [2,7]. 

According to the current analysis, Mashhad University of Medical Sciences, Shahid Beheshti University of Medical Sciences, the Ministry of Education of China, the Central Leather Research Institute of India, and the University of Sao Paulo are the top five institutions in terms of publications. The best network for collaboration, meanwhile, only includes Iranian institutions. Due to the relatively low level of collaboration across these institutes, initiatives to build deeper links may help this field advance in the future. More international cooperation would enhance information exchange and promote development in this area of study. However, among the top five researchers in terms of article counts, Meiyanto, Edy (ranked first with 9 publications) and Jenie, Riris (ranked third with 7 articles) are also the second most collaborating partners. More publications produced by authors demonstrate their high standing in the field, and collaboration between them would not only increase the likelihood that they may make in-depth findings about the wound-healing properties of curcumin but also aid to advance this subject in future. 

Through co-occurrence analyses, we sought to identify important research interests and topics in this field in order to aid researchers as they navigate through different studies. A co-occurrence network was generated based on the author’s as well as overall keywords. The most central and highly weighted keywords in this network are likely to represent research hotspots in this active field, with a further need for curcumin in wound-healing research pertaining to these topics and the associated research directions. Overlay visualization analysis revealed certain keywords with more research focus, suggesting them to be important active research areas in this field that warrant further study. In order to increase the bioavailability and biofunctionality of the molecule, curcumin is combined with a variety of delivery systems, the most popular of which are nanoparticle, biopolymer, nanofiber, and hydrogel [31,32,33,34]. Liposomal carrier, nanocapsule and micelles are some of the other drug delivery vehicles used with curcumin [32,33,34,35,36]. These findings also provide a basis for identifying active hotspots of scientific interest, which can be used to direct future research projects. 

## 3. Methodology

### 3.1. Data Extraction

In April 2022, we accessed the SCOPUS online database to identify publications with the search keyword “turmeric” OR “curcum*” AND “wound”. The identified publications that mentioned the word “curcumin” or “turmeric” or “curcuma spp.” and “wound” in the title, abstract, or keywords were selected for the bibliometric review analysis. The search results were limited to journal articles in English that were published until 2021. Citations of these articles collected up to January 2022 were considered. A total of 1460 articles appeared as a result. Then, manual screening was used to enhance the quality of the data by reading the entire texts and examining the content of the articles to filter out the unnecessary ones. When we decided whether an article should be included, we first considered its relevance to “curcumin as a wound-healing agent”. Consequently, 1284 journal articles were selected for the bibliometric analyses. Before proceeding to the analyses, the following factors were first used to evaluate the publications retrieved through the search: (1) Number of publications per year; (2) Organization; (3) Countries/Regions; (4) Journals; (5) Total Citations; and (6) Keywords. Afterwards, the names of authors and their corresponding institutions and countries/regions were extracted from the author addresses. Before importing the data into the VOSviewer software for analysis, authors with identical initials were searched independently and labeled with numbers.

For bibliometric studies, the complete records were downloaded and imported into the VOSviewer software. Bubble maps were produced and analyzed using default parameters. The font size of the words on the bubble map represents how frequently the terms occur (multiple appearances in a single publication count as one). If two words co-occurred in the evaluated articles more frequently, they are closer to one another. The most frequently used markers in bibliometric studies—article count, citation count and Hirsch index (H-index)—were taken into consideration to measure the influence and impact of the scientific research conducted. The academic level was also measured using the H-index from both a quality and a quantity perspective. 

### 3.2. Data Analysis

Using the VOSviewer software, the gathered data was analyzed [37,38]. On the subject of curcumin’s ability to treat wounds, 159 different journals published a total of 1284 articles from 98 different nations and 3658 different organizations. With a total of 43,739 citations and 16,208 keywords, these articles were contributed by 5503 authors. A trend analysis of publications each year is illustrated, displaying the pattern of articles published from 1942 to 2021. The co-occurrence of total keywords, author’s keywords and co-authorship analysis was performed by using the full counting method showing only the elements connected with each other. Different visualization maps representing the collaboration network of authors, nations, and organizations were created where larger circles indicate more collaborations and stronger links between those who collaborate more frequently. The keyword map with full counting method assigns equal weight to each co-occurrence link and clusters related keywords together such that the circles around the terms that occur more frequently are larger. 

## 4. Conclusions

The present study demonstrated the advancements in scientific knowledge on curcumin and wound healing between 1942 and 2021, based on the literature found in the SCOPUS database. By identifying key contributors and outlining the scientific collaborations, the results represent a broad overview of the literature already available. We also identified trending keywords and the most researched topics using keyword co-occurrence analysis and overlay visualization. The results collected can serve as a guide for those wanting to make contributions to this field by providing details about active journals, authors, and widely researched topics in this area. As evidence of newly introduced drug-delivery strategies such as electro-spinning techniques, hydrogels, biomechanics, nanopharmaceutics, emulsion, nanofibres, etc., the amount of literature on the topic has doubled since 2018. This is due to new drug formulation methods and incorporation of bioengineering and biotechnology approaches. The present bibliometric review on the application of curcumin for wound healing not only highlights the present research paths, but also suggests that different drug-delivery mechanisms could be possible in the future, increasing the molecule’s bioavailability and advancing this particular area of study.

## Figures and Tables

**Table 1:** Top 10 journals ranked by article count.

Journal	Article Count	Citation Count	Average Citation per Article (ACPA)	H-Index	Most Cited Article	Times Cited
International Journal of Biological Macromolecules	44	2012	45.72	144	A review on chitosan and its nanocomposites in drug delivery	489
Journal Of Ethnopharmacology	28	1231	43.96	205	Ferula asafoetida and Curcuma longa in traditional medical treatment and diet in Nepal	220
Current Pharmaceutical Design	20	812	40.6	166	A review of therapeutic effects of curcumin	212
Materials Science and Engineering C	20	714	35.7	145	Antibacterial performance and in vivo diabetic wound healing of curcumin loaded gum tragacanth/poly(ε-caprolactone) electro spun nanofibers	153
International Journal of Pharmaceutics	14	529	31.11	229	In situ injectable nano-composite hydrogel composed of curcumin, N,O-carboxymethyl chitosan and oxidized alginate for wound healing application	159
Journal Of Drug Delivery Science and Technology	14	297	21.21	52	Curcumin-loaded electro spun PHBV nanofibers as potential wound-dressing material	83
International Journal of Molecular Sciences	13	664	51.07	195	Targeting the redox balance in inflammatory skin conditions	271
Phytotherapy Research	13	473	36.38	140	Radioprotection by plant products: Present status and future prospects	307
Biomedicine And Pharmacotherapy	10	219	21.9	109	Antidotal or protective effects of Curcuma longa (turmeric) and its active ingredient, curcumin, against natural and chemical toxicities: A review	49
International Journal of Pharmacy and Pharmaceutical Sciences	10	79	7.9	49	Documentation of ethnomedicinal knowledge of hilly tract areas of East Godavari District Of Andhra Pradesh, India	13

**Table 2:** Top 10 countries ranked by article count.

Country	Article Count	Total Citations	Average Citation per Article (ACPA)
India	354	11,361	32.09
China	240	6346	26.44
USA	164	13,361	81.46
Iran	111	3068	27.63
Italy	45	1867	41.48
Indonesia	40	191	4.77
Pakistan	40	907	22.67
Thailand	39	555	14.23
Malaysia	38	712	18.73
South Korea	36	1615	44.86

**Table 3:** Top 10 authors from an article count perspective.

Authors	Article Count	Total Citations	Average Citation per Article
Meiyanto, Edy	9	51	5.6
Sahebkar, Amirhossein	8	192	24
Jenie, Riris	7	51	7.2
Hussain, Zahid	6	310	51.6
Meizarini, Asti	6	29	4.8
Ramakrishna, Seeram	6	184	30.6
Suwantong, Orawan	6	124	20.6
Aggarwal B.B.	5	2891	578.2
Amini, Abdollah	5	46	9.2
Maheshwari R.K.	5	721	144.2

**Table 4:** Top 10 Institutions ranked by their article count.

Organization	Article Count	Total Citations	Average Citation per Article
Mashhad University of Medical Sciences	66	1640	24.84
Shahid Beheshti University of Medical Sciences	32	535	16.71
Ministry of Education China	23	329	14.30
Central Leather Research Institute India	21	1328	63.23
Universidade de São Paulo	16	504	31.5
Sichuan University	13	719	55.30
Tehran University of Medical Sciences	13	611	47
Iran University of Medical Sciences	13	271	20.84
Tabriz University of Medical Sciences	13	343	26.38
Amirkabir University of Technology	12	432	39.27

**Table 5:** Top 20 keywords with their occurrences.

Total Keywords	Co-Occurrence	Author’s Keyword	Co-Occurrence
curcumin	922	curcumin	429
wound healing	702	wound healing	210
review	281	wound dressing	56
animal experiment	258	chitosan	48
cell proliferation	251	electrospinning	46
antioxidant activity	188	inflammation	41
drug delivery system	186	antioxidant	37
anti-inflammatory activity	183	turmeric	35
animal tissue	181	apoptosis	34
curcuma longa	171	nanoparticles	34
cell migration	170	curcuma longa	33
antineoplastic activity	169	anti-inflammatory	31
plant extract	154	drug delivery	29
inflammation	144	collagen	27
antibacterial activity	135	migration	27
chitosan	135	medicinal plants	26
drug efficacy	135	angiogenesis	25
antioxidant	130	antibacterial	23
biocompatibility	130	invasion	22
nanoparticle	122	wound	22

## Data Availability

Not Applicable.
